# Dye Decolorization by a Miniaturized Peroxidase Fe-MimochromeVI*a

**DOI:** 10.3390/ijms241311070

**Published:** 2023-07-04

**Authors:** Marco Chino, Salvatore La Gatta, Linda Leone, Maria De Fenza, Angela Lombardi, Vincenzo Pavone, Ornella Maglio

**Affiliations:** 1 Department of Chemical Sciences, University of Napoli Federico II, Via Cintia, 80126 Napoli, Italy; salvatore.lagatta@unina.it (S.L.G.); linda.leone@unina.it (L.L.); maria.defenza@unina.it (M.D.F.); alombard@unina.it (A.L.); vincenzo.pavone@unina.it (V.P.); 2 Institute of Biostructures and Bioimaging (IBB), National Research Council (CNR), Via Pietro Castellino 111, 80131 Napoli, Italy

**Keywords:** peroxidase, artificial metalloenzyme, bleaching, dye degradation, enzymatic treatment

## Abstract

Oxidases and peroxidases have found application in the field of chlorine-free organic dye degradation in the paper, toothpaste, and detergent industries. Nevertheless, their widespread use is somehow hindered because of their cost, availability, and batch-to-batch reproducibility. Here, we report the catalytic proficiency of a miniaturized synthetic peroxidase, Fe-Mimochrome VI*a, in the decolorization of four organic dyes, as representatives of either the heterocyclic or triarylmethane class of dyes. Fe-Mimochrome VI*a performed over 130 turnovers in less than five minutes in an aqueous buffer at a neutral pH under mild conditions.

## 1. Introduction

Bleaching processes are widespread at different levels, with bleaching agents being adopted and/or sold by companies and institutions, as well as end users. Their fields of application are spread out over several markets, from laundry to paper and pulp industries, passing through leather, cosmetics, and pharma industries. The total revenues of the bleaching market are difficult to estimate, given its intrinsic cross-sectional features. We could just consider, for example, that, each day, more than ten thousand tons of hydrogen peroxide, one of the most widely used bleaching agents, is consumed worldwide.

Bleaching in solution and/or on surfaces consists of the discoloring (whitening) process that occurs upon the oxidation of any given dye. Most common dyes (and stains) usually contain chromophores, such as aromatic rings and conjugated systems, that can absorb visible light. These chromophores, when oxidized, shift their absorption wavelength, making them invisible to the human eye. Chlorine-based bleaches are among the most common bleaches used worldwide. However, as a consequence of their large use, safety concerns have been raised about the formation and release in the environment of organochlorines, dioxins, and chlorinated volatile organic compounds, some of them being potential human carcinogens [1]. Peroxide-based bleaches are less efficient than chlorine-based; nevertheless, they constitute a health hazard to a much lower extent. For this reason, both academic and industrial research focuses on improving peroxide-based bleaching efficiency and selectivity. In general, bleaching by hydrogen peroxide occurs thanks to the formation of different active oxygen species, depending on the reaction conditions (temperature, pH, light, and presence of transition metals). In the presence of transition metal catalysts, highly oxidizing species are produced, which, in turn, based on their molecular properties, perform the specific oxidation of substrates. Nature exploits metalloenzymes, particularly peroxidases, in the activation of hydrogen peroxide. Such metalloenzymes are very attractive for practical applications in chemistry, biotechnology, and medicine for their great versatility, and they have been already implemented in the degradation process of various substrates [2,3,4,5,6]. Examples of noticeable applications are: decontamination of environmental pollutants [7,8], delignification in the paper industry [9,10], diagnostic kit development [11], immunoassay [12,13], organic and polymer synthesis [14,15], biosensor technology [16,17], determination and quantification of hydrogen peroxide [18,19], decolorization of industrial effluents [20,21,22,23], and tooth whitening [24,25]. Although peroxidase enzymes are now quite spread out both in commercial products and industrial processes, their full applicability has some limitations. The main problems arise from the relatively low stability and activity of such enzymes at high temperatures, extreme pH ranges, high salt concentrations, and organic solvents, as well as poor batch-to-batch reproducibility. Therefore, the engineering of improved peroxidases is highly desirable [26,27,28,29,30,31,32,33]. Among them, building artificial “custom-made” enzymes, able to mimic natural peroxidases and optimized for a specific application, seems highly convenient [34,35,36,37,38].

**Figure 1:**
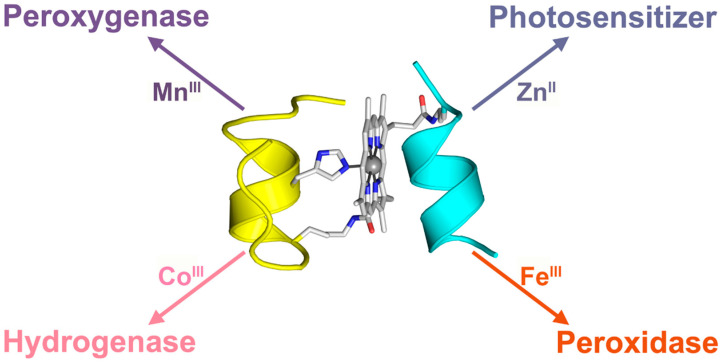
MC6*a designed model and its dominant enzymatic activities exhibited with different metals.

Recently, we prepared a fully synthetic miniaturized peroxidase, namely Fe^III^–mimochrome VI*a (FeMC6*a), able to mimic naturally occurring peroxidases, with catalytic performances comparable to natural ones [36]. FeMC6*a is able to activate hydrogen peroxide to perform 2,2′-azino-bis(3-ethylbenzothiazoline-6-sulfonic acid (ABTS) oxidation [39], thioanisole oxygenation [40], luminol oxidation for sensing purposes [41,42], and the dehalogenation and polymerization of halogenated phenols [43,44]. Besides oxidation, the metal replacement in MC6*a leads to significant shifts in the reactivity. Indeed, a manganese complex is a competent catalyst in the selective monooxygenation of thioanisole and indoles [40,45], the cobalt derivative is active as the hydrogen evolution catalyst [46,47,48], and the zinc one is suitable as a photosensitizer [49] (Figure 1).

In this study, we show that FeMC6*a displays a remarkable activity in the decolorization of four dyes: Neutral Red (NR), Methylene Blue (MB), Xylenol Orange (XO), and Bromophenol Blue (BPB).

## 2. Results and Discussion

### 2.1. Preliminary Screening of Dye Activity

**Figure 2:**
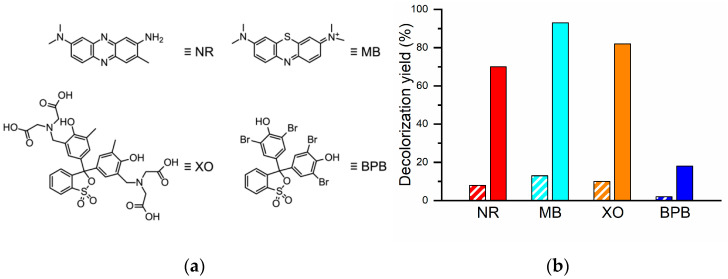
Structures of the dyes tested in this work. (**a**) Neutral Red (NR), Methylene Blue (MB), Xylenol Orange (XO), and Bromophenol Blue (BPB) are shown in one representative resonance/tautomeric structure over several possible ones. The bar plot (**b**) reports the decolorization percentage for NR (110 μM, red), MB (12.9 μM, cyan), XO (78 μM, orange), and BPB (13.5 μM, blue) when 1 μM FeMC6*a was absent (left, half filled) or present (right, filled) in the reaction mixture (100 mM phosphate buffer, pH 6.5, 0.25 mM H_2_O_2_).

To assess the performance of the artificial FeMC6*a peroxidase in dye-bleaching applications, using hydrogen peroxide as the oxidant, we selected four organic dyes, including two heterocyclic dyes (NR and MB, Figure 2a) and two triarylmethane dyes (XO and BPB, Figure 2a). NR is a phenazine dye that displays a deep red color at pH values below 6.6, whereas MB is a thiazine dye displaying a cyan-like color in its oxidized form. BPB is a brominated dye with a deep blue color at pH values above 4.5, and XO is a pH indicator that displays colorations ranging from deep purple to light orange based on pH values. The investigation employed very mild conditions to serve as the basis for an environmentally sustainable decolorizing treatment process.

In particular, catalytic assays were performed at an almost neutral pH in a mild saline buffer without any organic cosolvent (100 mM sodium phosphate buffer, pH 6.5), using a cost-effective amount of the catalyst (1 μM) and 250 molar excess of the environmental-friendly oxidant hydrogen peroxide (0.25 mM). The substrate concentrations were different for each dye and mostly dependent on their extinction coefficients (see Section 3 and Table S1 in the Supplementary Materials).

FeMC6*a displayed functional proficiency towards this panel of substrates under the evaluated conditions, as evidenced by the significant decolorization percentage (see Section 3) with respect to the reactions performed in the absence of the catalyst (Figure 2b).

In detail, FeMC6*a exhibited excellent activity against MB and XO with decolorization percentages above 80% and good activity against NR, with a decolorization percentage of 70%. Conversely, only modest activity (18%) was observed in the presence of BPB. A negligible reaction progress (decolorization < 10%) was observed for the uncatalyzed oxidation, thus proving the involvement of FeMC6*a in driving the dye oxidation. 

**Figure 3:**
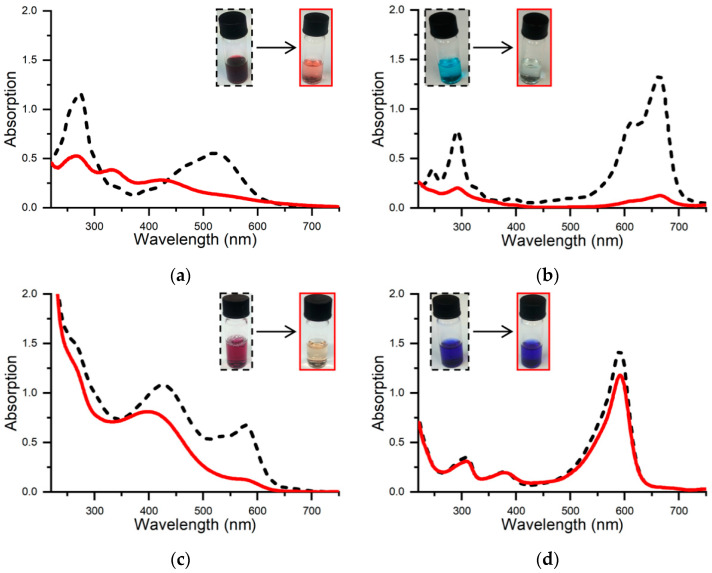
Decolorization process as followed by UV–Vis spectroscopy. Two spectra are reported for each dye, the first before the addition of H_2_O_2_ (black dashed line) and the second after the addition of H_2_O_2_, when no further consumption of the dye was observed (red line). All the reactions were performed in a solution of the dye and 1 μM FeMC6*a in 100 mM phosphate buffer, pH 6.5, to which 0.25 mM H_2_O_2_ was added. Dye oxidation was monitored over 15 min for NR (60 μM, (**a**)), MB (12.9 μM, (**b**)), XO (78 μM, (**c**)), and BPB (13.5 μM, (**d**)). Insets show the photographic pictures of the reaction mixtures before (**left**) and after (**right**) decolorization.

The UV–Visible spectral changes were analyzed to get further insight on the catalyst performance (Figure 3). FeMC6*a was remarkably fast in performing dye decolorization under the preliminary experimental conditions. Oxidation of the dark red solution of NR led to a light pink solution in just 5 min, and no significant change was subsequently observed (Figure 3a and Figure S1). Interestingly, two bands appeared around 330 and 430 nm in the final spectrum, responsible for the final nuance of the solution.

The characteristic absorbance of MB at 663 nm decreased after mixing H_2_O_2_, and the blue solution turned colorless (A_663_ < 0.5) within 3 min (Figure 3b and Figure S2). Besides some contribution of the remaining MB, no oxidation product could be detected in the visible region of the final spectrum. The decolorization of XO, monitored at 580 nm, slowly led to a light orange solution from a brilliant purple after 15 min (Figure 3c). The final UV spectrum, featuring a broad band centered around 400 nm, showed a hypochromic shift of the XO 425 nm maximum. Finally, FeMC6*a did not exhibit a remarkable activity against BPB. Indeed, after 5 min of reaction, the dye decolorization was only 18%, and no further change was observed (Figure 3d). 

Table 1 reports the efficiency of FeMC6*a in comparison with previous dye decolorization procedures that were carried out by either enzymatic or nonenzymatic methodologies [50,51] (Table 1). 

It is worth noting here that no satisfactory enzymatic degradation treatment has been reported to date for NR, with the exception of a fungal treatment performed with *Perenniporia subacida* under acidic conditions (~97%, 10-day incubation period) [52]. Moreover, a modest decolorizing efficiency of ~33% was obtained for NR after only 24 h of treatment with a fungal laccase [53]. Interestingly, the highly promiscuous DyP was not proficient in NR oxidation when 50 μM of NR was reacted with 100 μM of hydrogen peroxide. Nevertheless, a nonenzymatic treatment with a Fenton catalyst, under acidic conditions, gave a very good decolorization percentage in a short timeframe (98% decolorization percentage in <5 min). 

**Table 1:** Maximum decolorization percentages and reaction time comparison for the four dye objects of this work.

Substrate	FeMC6*a	HRP ^†^	SBP ^†^	LiP ^†^	*Tfu*DyP ^†^	Lac ^†^	Inorganic Catalyst
NR	70% 5 min	-	-	-	0% ^a^ 1 h	33% ^b^ 24 h	84% ^c^; 98% ^d^ 30 min; <5 min
MB	95% 3 min	21% ^e^ 60 min	-	85% ^f^ 15 min	0% ^a^ 1 h	0% ^b^ 24 h	90% ^g^; 96% ^h^ 2 h; 1 h
XO	82% 15 min	-	-	-	-	-	>95% ^i^; 42% ^h^ 45 min; 1 h
BPB	18% 5 min	95% ^j^ 10 min	85% ^k^ 60 min	93% ^l^ 15 min	-	14% ^b^ 24 h	90% ^m^ 25 min

^†^ HRP—horseradish peroxidase; SBP—soybean peroxidase; LiP—lignin peroxidase; DyP—dye-decolorizing peroxidase from *Thermobifida fusca*; Lac—laccase isoform *Lac2* from *P. Nebrodensis*. ^a^ pH range 3.0–5.0 [54]. ^b^ pH = 4.0, ABTS as the redox mediator [53]. ^c^ Photo-oxidative process with H_2_O_2_. ^d^ Fenton process [55]. ^e^ pH = 3.0, T = 30 °C [56]. ^f^ pH = 2.5 crude lignin peroxidase from a culture medium of *P. Chrysosporium* [57]. ^g^ pH = 3, Mn_3_O_4_ nanoparticles [58]. ^h^ Lignin peroxidase-like biomimetic catalyst [59]. ^i^ Catalytic porous Fe_3_O_4_ nanospheres and H_2_O_2_ as the oxidant [60]. ^j^ pH = 6 [61]. ^k^ pH = 3, 5, and 7, immobilized-aminated peroxidase [62]. ^l^ pH = 4.0, crude lignin peroxidase from a culture medium of *P. Chrysosporium* [57]. ^m^ pH = 7.0, photocatalytic process with photocatalysts ZnO and Ag^+^-doped ZnO [63].

FeMC6*a showed comparable results in terms of the decolorization yield and reaction times when LiP was used as the catalyst for MB oxidation (85% decolorization yield in 15 min) under similar experimental conditions [57]. The enzymatic treatment, using HRP as the catalyst [56], gave a lower dye-decolorizing effect (21%) and higher reaction times (>60 min). Such an efficiency difference may be ascribed to the higher reduction potentials of the Compound I/Compound II and Compound II/Fe^3+^ pairs in LiP, being roughly double the HRP ones [64,65]. A remarkable dye-decoloring effect (>90%) was also achieved for the removal of MB using chemical or physical methods after reaction times ranging from 60 to 120 min [58,59].

Unlike other dyes, enzymes are still unemployed to decolorize XO. Porous Fe_3_O_4_ nanospheres were used as the catalyst for the degradation of Xylenol Orange (XO) in an aqueous solution with H_2_O_2_ as the oxidant. This system was able to degrade XO up to 95% in 45 min [60], only a slightly higher efficiency than FeMC6*a. A LiP mimic catalytic system, consisting of an immobilized Mn^III^-tetrakis(4-sulfonato-phenyl)porphyrin, has been also tested to perform XO degradation. However, only 42% was degraded by the addition of 8.8 mM of H_2_O_2_ when this alternative peroxidase mimic was used [59].

Finally, three out of the four peroxidases here compared are amenable for BPB degradation (Table 1). A similar reactivity was expected for FeMC6*a, which has already been shown proficient in 2,4,6-trichlorophenol (TCP) dehalogenation [43]. However, only a modest efficiency was observed towards BPB under our experimental conditions, as previously mentioned. It is worth noticing that a striking 200-fold increase in the catalytic efficiency was observed when the dehalogenation of TCP was performed in the presence of trifluoroethanol (TFE) as the cosolvent [43]. It has been previously shown that TFE induces the sandwiched structure in MC6*a, driving the positioning of the distal peptide on the heme [39], and, in turn, this can be correlated with the observed overpotential shift in hydrogen evolution for CoMC6*a [46]. Therefore, TFE may be critical in the oxidation of halophenols, thus explaining the poor performance here observed.

### 2.2. Optimization of Experimental Conditions

Given the more promising results on heterocyclic dyes, a deeper optimization of the experimental conditions was performed as a function of catalyst, peroxide, and substrate concentrations. The purpose was to find the minimal amount of catalyst and peroxide that must be added to keep the highest decolorization percentage. To perform a full concentration screening, a plate reader was used to register the spectra under different conditions in a 24-well plate each minute for 5 min. 

**Figure 4:**
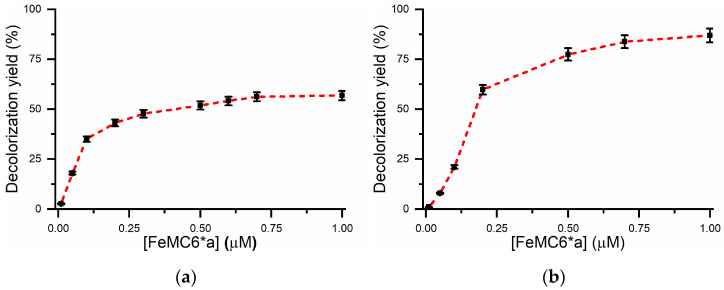
Decolorization percentage of NR (110 μM (**a**)) and MB (9.9 μM (**b**)) as a function of the FeMC6*a concentration. The reaction was performed in 100 mM phosphate buffer, pH 6.5, 3 mM H_2_O_2_. The dashed line between each point was intended as a guide for the eye, while the error bars defined a 10% confidence level.

We started by investigating the effect of the catalyst concentration on NR and MB decolorization in the range 10 nM–1.0 μM (Figure 4). 

The maximum decolorization efficiency could be obtained at a 1.0 μM FeMC6*a concentration for both dyes, where 57% and 87% decolorization were achieved for NR and MB, respectively. Nevertheless, a comparable catalytic performance could be obtained at a lower FeMC6*a concentration (0.7 μM). 

**Figure 5:**
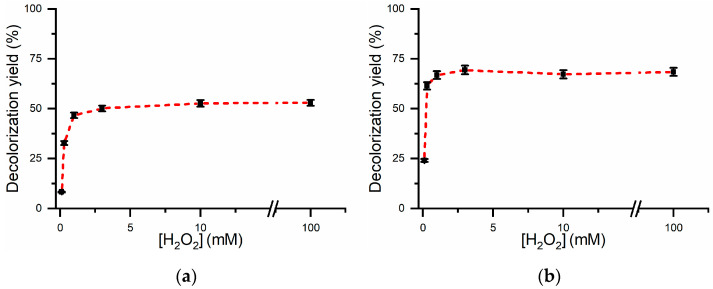
Decolorization percentage of NR (110 μM (**a**)) and MB (9.9 μM (**b**)) as a function of the H_2_O_2_ concentration. The reaction was performed in 100 mM phosphate buffer, pH 6.5, 0.5 μM FeMC6*a. The dashed line between each point was intended as a guide for the eye, while the error bars defined a 10% confidence level.

Next, the effect of the peroxide concentration was investigated in the range 0.1–100 mM. The decolorization percentage as a function of the H_2_O_2_ concentration was steeply saturated around 3.0 mM for both dyes (Figure 5). 

Indeed, for NR and MB, the highest percentages of conversion observed were 53% and 70%, respectively. However, the results obtained with any peroxide concentration higher than 1.0 mM showed very similar results. Further, the reaction performed with a peroxide concentration as low as 0.1 mM showed performance levels close to the uncatalyzed reaction. 

**Figure 6:**
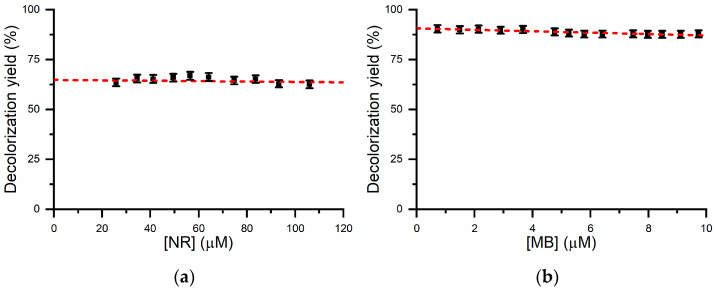
Decolorization percentage of NR (**a**) and MB (**b**) as a function of their respective concentrations (26–106 μM and 0.72–10 μM, respectively). The reaction was performed in 100 mM phosphate buffer, pH 6.5, 0.5 μM FeMC6*a, and 3.0 mM H_2_O_2_. The dashed line between each point was intended as a guide for the eye, while the error bars defined a 10% confidence level.

Lastly, the effect of the substrate concentration was screened. The concentration ranges were different for the two substrates due to their different extinction coefficients (in order to avoid detector saturation in the spectrophotometer) and different aggregation propensity of the substrates. Therefore, the NR and MB concentrations were screened in the ranges of 26–106 μM and 0.72–10 μM, respectively (Figure 6).

The plot clearly indicates that, in the evaluated range, both NR and MB concentrations have a negligible effect on the yield of the reaction. In detail, the decolorization percentage ranges from a maximum of 67% to a minimum of 63% for NR, and from 91% to 87% for MB, in 5 min. A rough estimation of the turnover number (TON) can be calculated from the decolorization percentage, corresponding to 134 and 17 turnovers, respectively. Therefore, FeMC6*a can perform several TONs under the explored concentration ranges without any significant bleaching of the catalyst.

Overall, our analysis defines that a mildly oxidizing solution of 3 mM H_2_O_2_ in phosphate buffer at pH 6.5 is able to oxidize at least either 106 or 10 μM of NR and MB, respectively, by using an amount of FeMC6*a as low as 0.5 μM in the concentration (1.75 mg/L).

### 2.3. Estimation of the Catalytic Efficiency for Neutral Red Oxidation

**Figure 7:**
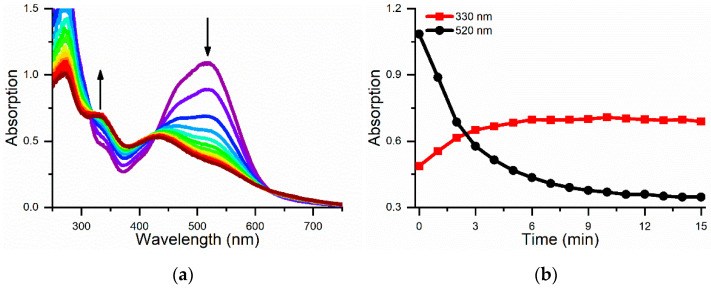
NR (0.12 mM) degradation followed by UV–Vis absorption spectroscopy. Spectra were acquired every minute for 15 min (colored lines from violet to red) (**a**). The absorption band decrease at 520 nm is accompanied by the formation of a band at 330 nm (**b**). The reaction was performed in 100 mM phosphate buffer, pH 6.5, 1.0 μM FeMC6*a, and 0.10 mM H_2_O_2_.

As previously mentioned, NR degradation is accompanied by the appearance of two bands around 330 and 430 nm. These bands are characterized by a lower extinction coefficient with respect to the NR absorption features at 280/520 nm and can be most probably related to the formation of a single oxidation product. To better understand this phenomenon, the NR oxidation catalyzed by FeMC6*a was studied in further detail. NR oxidation was followed every minute for 15 min in the presence of a lower amount of hydrogen peroxide (0.10 mM) and with a slightly higher concentration of substrate (0.12 mM, Figure 7).

Two isosbestic points at 443 nm and 308 nm confirmed the preliminary hypothesis that FeMC6*a stoichiometrically converts NR to a single product (Figure 7a). NR was previously oxidized by unselective Fenton chemistry or by photooxidation [55,66]; therefore, only limited data has been acquired so far about product identities. In one case, demethylation of the ternary amine, as well as deamination, were invoked after photooxidation; however, no UV spectra were reported for these products. From a different perspective, NR, as well as other phenazines, is able to perform oxidative polymerization on the surfaces of electrodes (deposition potential 0.9–1.2 V vs. SHE) [67,68,69,70]. In both cases, relatively stable radical species should be involved in the mechanism of polymerization. In our case, a partial loss of conjugation might be expected based on the UV band shifts, thus supporting radical cation formation, but unfortunately, at the moment, the identity of the elusive oxidation product could not be defined.

**Figure 8:**
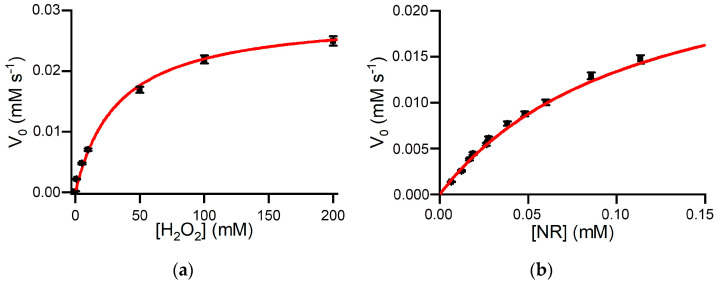
Oxidation activity of FeMC6*a. (**a**) Initial rate dependence towards the H_2_O_2_ concentration. The reaction conditions were FeMC6*a (1 µM) and NR (25 mM) in 100 mM phosphate buffer, pH 6.5. (**b**) Initial rate dependence towards NR concentration. The reaction conditions were FeMC6*a (1 µM) and H_2_O_2_ (0.10 M) in 100 mM phosphate buffer, pH 6.5. The data points were fitted using the Michaelis–Menten equation (red lines), and the error bars corresponded to the standard deviation of three repetitions.

Interestingly, NR oxidation by FeMC6*a involves the formation of a single product; thus, the reaction is amenable for a deeper catalytic characterization. Two sets of kinetic experiments were performed by varying the H_2_O_2_ concentration at a fixed NR concentration and vice versa (Figure 8). 

**Scheme 1:**
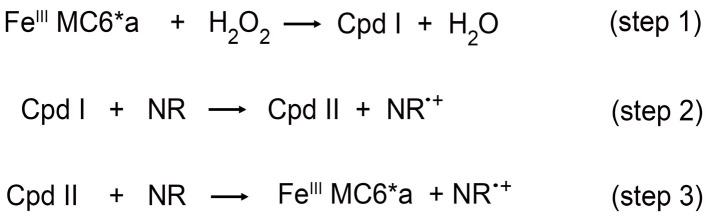
Proposed mechanism for NR oxidation by FeMC6*a.

The initial rates of NR oxidation (v_0_) were plotted as a function of both substrate concentrations by keeping a high excess of either NR (25 mM, Figure 8a) or H_2_O_2_ (0.1 M, Figure 8b). The enzyme activity follows the typical Michaelis–Menten kinetics in both cases. Therefore, as previously reported for other substrates, a reaction mechanism might be invoked (Scheme 1) that involves the formation of two highly oxidizing iron-oxo intermediates, the so-called Compound I (Cpd I) and Compound II (Cpd II). 

The catalytic parameters were determined by fitting v_0_ values with a simple single-substrate equation (Table 2).

A direct comparison of the *k*_cat_ value for NR oxidation to that obtained for TCP, under the same experimental conditions, reveals that FeMC6*a performs approximately half the turnovers per second [43]. This is also directly reflected on the kinetic efficiency (*k*_cat_/K_m_), given that the K_m_ values are equivalent within the experimental error. The *k*_cat_ values have been previously found to be inversely proportional to the reduction potentials of the substrates [71,72]. This is actually reflected in the lower reduction potential of TCP (~0.9 V vs. SHE) with respect to NR (~1.2 V vs. SHE) [70,72]. Finally, when compared to other dye-degrading peroxidase mimics, the FeMC6*a turnover frequency values are fairly similar [28,72].

It is worth noting that K_m_^H_2_O_2_^ is significantly lower than previously assessed, indicating that FeMC6*a has a higher affinity for peroxide during NR oxidation. Two possible explanations may be proposed. The former involves the formation of a NR/FeMC6*a complex that is apparently more active towards peroxide than FeMC6*a alone, most probably by drifting the iron reduction potential. The latter may be attributed to an active role of NR in the protonation/deprotonation steps along the reaction pathway. Nevertheless, both explanations may concur with the observed outcome.

## 3. Materials and Methods

All reagents used were purchased from Merck (Merck KGaA, Darmstadt, Germany) and used without further purification. All organic solvents used were supplied by Romil (Cambridge, UK). Phosphate salts (monobasic and dibasic) for preparation of the buffers and H_2_O_2_ (30% *v*/*v*) were provided by Merck. All buffer solutions were made by using water with a HPLC purity grade (Romil). Data analysis was performed using OriginPro, version 9.0 (Origin Lab Corporation, Northampton, MA, USA). All chromatographic analyses and purifications were performed using HPLC grade solvents. All stock solutions were stored at 4 °C away from light, unless otherwise specified. The UV–Vis analysis and kinetic experiments were recorded with a Cary 60 spectrophotometer (Agilent, Santa Clara, CA, USA) equipped with a thermostatic cell compartment, using quartz cuvettes with 0.10, 0.01, and 1.00 cm path lengths. Wavelength scans were performed at 25 °C from 200 to 800 nm, with a 600 nm min^−1^ scan speed. All data were blank-corrected.

Fe-MC6*a was synthesized, purified, and characterized as previously described by us [39].

Stock solutions of Fe-MC6*a were prepared by dissolving the pure, lyophilized compound in acidic water (0.1% TFA *v*/*v*) and diluted to the final concentration in the reaction buffer (100 mM sodium phosphate, pH 6.5). Concentrations were determined spectrophotometrically using a molar extinction coefficient (ε_387_) of 1.7 × 10^5^ M^−1^cm^−1^. Stock solutions of H_2_O_2_ were prepared by the proper dilution of a commercial stock solution (30% *w*/*w* in H_2_O), and their concentration was determined by UV–Vis absorption spectroscopy using ε_240_ = 39.40 M^−1^ cm^−1^.

Stock solutions of the dyes were freshly prepared by dissolving a weighted amount of the pure compound in water. The concentration was checked spectrophotometrically using the determined molar extinction coefficient (see Table S1 and Figures S3–S6 in the Supplementary Materials). Especially for MB, the maximum at 663 nm of the monomeric species was used to determine the final concentration, because the band at 610 nm has been previously attributed to the dimeric species [73]. 

### 3.1. Preliminary Screening of Dye Activity

The FeMC6*a catalytic efficiency in dye-decolorizing and substrate degradation has been explored by monitoring the changes in the absorption spectra every minute for a total of 60 min using the following operational conditions: 100 mM sodium phosphate buffer, pH 6.5, 1 μM catalyst concentration, and 0.25 mM peroxide concentration. The following substrate concentrations were used: Bromophenol Blue 1.35 × 10^−5^ M, Methylene Blue 1.29 × 10^−5^ M, Neutral Red 0.60 × 10^−4^ M, and Xylenol Orange 7.80 × 10^−5^ M.

The dye decolorization rate was estimated by measuring the absorbance of the different dyes at the following wavelengths: λ = 520 nm for NR, λ = 663 nm for MB, λ = 591 nm for BPB, and λ = 580 nm for XO. The decolorizing yield was calculated using the following Equation (1):

(1) $$Decolorization(\%)=\frac{\left(A_{i}-A_{f}\right)}{A_{f}\times 100}$$

where A_i_ and A_f_ are the initial and the final absorbance of the dyes, respectively.

### 3.2. Optimization of Experimental Conditions for Catalytic Activity of FeMC6*a

The experimental conditions for the catalytic assays were optimized in terms of the catalyst and substrate concentrations. The optimization was performed using the Tecan Spark plate reader (Tecan Trading AG, Männedorf, Switzerland) with Corning 24-well Clear Multiple Well Plates as the reaction vessels. Each well was filled to a final volume V = 2.0 mL to gain a path length of 1.0 cm. 

The experiments were performed at variable catalyst concentrations (in the range of 0.1 nM–1.0 μM) by using a fixed concentration of the substrate (9.9 μM and 0.11 mM for MB and NR, respectively) and of hydrogen peroxide (3.0 mM). 

The reactions were started by the addition of H_2_O_2_ to a stirring mixture of catalyst preloaded with the substrate, and the reaction progress was followed over five minutes by monitoring the changes in the absorbance band at λ = 663 nm for MB and λ = 520 nm for NR. The absorption values were recorded every minute.

To evaluate the optimum H_2_O_2_ concentration, the catalyst and the substrate concentrations were fixed while different H_2_O_2_ concentrations (0.1–20 mM) were explored.

In these experiments, the FeMC6*a concentration was 0.5 μM, and the substrate concentration was 9.9 μM and 110 μM for MB and NR, respectively. 

Finally, the effect of the substrate concentration was investigated. The experiments were performed at a 0.5 μM concentration of FeMC6*a and a 3.0 mM concentration of oxidizing substrate, while the substrates were varied in the range of 0.72–10 μM for MB and from 26 to 106 μM for NR.

### 3.3. Catalytic Assays

Kinetic assays were performed using an Agilent Cary 60 spectrophotometer equipped with a thermostated cell holder and magnetic stirrer. The kinetic measurements were performed at 25 °C and under magnetic stirring, using quartz cuvettes with a 1.0 cm path length. The dye used in the catalytic assay was Neutral Red. To evaluate the enzyme activity, the decreasing of the absorbance at λ = 520 nm (ε_520_ = 9.47 × 10^3^ M^−1^ cm^−1^) was monitored for 10 min.

All reactions were carried out in phosphate buffer (100 mM, pH 6.5). The enzyme concentration in all the experiments was fixed at 1.00 μM. The stock solution of FeMC6*a was prepared in H_2_O 0.1% (*v*/*v*) TFA (ε_387_ = 1.17 × 10^5^ M^−1^ cm^−1^) and diluted to the final concentration (1.00 μM) in the reaction buffer. 

The substrate and hydrogen peroxide stock solutions were freshly prepared, and their initial concentrations were determined by UV–Vis spectroscopy (H_2_O_2_, ε_240_ = 39.4 M^−1^ cm^−1^).

The K_m_ values for NR and H_2_O_2_ were measured by keeping constant one of the two substrate concentrations while varying the other one and vice versa. The progress curves of the reaction were monitored at λ = 520 nm. For the determination of K_m_^H_2_O_2_^, the NR concentration was fixed at 25 mM, while H_2_O_2_ was varied from 0 to 200 mM. For the determination of K_m_^NR^, the H_2_O_2_ concentration was fixed at 100 mM, while the NR concentration was varied from 0 to 110 μM.

The initial rate (v_0_, mM s^−1^) values were plotted as a function of the substrate concentrations, and the data points were fitted according to the Michaelis–Menten equation using Origin Pro 9.0 software.

## 4. Conclusions

In this work, miniaturized peroxidase FeMC6*a was employed as the catalyst for the decolorization of four dyes: two heterocyclic dyes (Neutral Red, NR and Methylene Blue, MB) and two triarylmethane dyes (Xylenol Orange, XO and Bromophenol Blue, BPB). The results obtained with FeMC6*a look very promising compared to those reported in the literature for all four dyes. 

In particular, the enzymatic reaction catalyzed by FeMC6*a in the presence of H_2_O_2_ allowed oxidation, with good yields and within a short time range (3–15 min), of three out of the four dyes screened in this study. 

Our catalyst is able to perform dye decolorization at an almost neutral pH under mild experimental conditions in the absence of organic cosolvents and using a green and environmentally friendly oxidant. Notably, a remarkable dye-decoloring effect was observed for two dyes, namely XO and NR, for which enzymatic treatments were not reported or unsatisfactory. In particular, differently from DyP, FeMC6*a is capable of oxidizing NR, most probably because of its very high potential. Further, our catalyst displayed catalytic efficiency comparable to those reported for other dye-degrading peroxidase mimics. An intriguing aspect to be explored is represented by the combined use of the FeMC6*a/H_2_O_2_ bleaching system in combination with photocatalytic nanomaterials as a way to generate a hybrid photocatalyst [74]. By combining the selective oxidative processes of the synthetic enzyme with the photoreductive capabilities of such nanomaterials, a wider range of dyes could be degraded, hopefully with high efficiency.

All the results reported herein demonstrated that FeMC6*a is a promiscuous peroxidase with a wide substrate specificity that could be employed in the area of catalytic bleaching, an active industrial field. A specific instance comes from the leather industry, for which there is an urgent need for cheap but selective bleaching agents capable of preventing Cr(III) to Cr(VI) oxidation, a life-threatening pollutant. This practical application could have a considerable economic impact, due to the urgency of preserving and protecting the environment. The use of our miniaturized peroxidase could also provide new opportunities for the synthesis of value-added products from pollutants towards a circular economy.

## Figures and Tables

**Table 2:** Comparison of the Michaelis–Menten parameters for FeMC6*a.

Substrate (S)	*k*_cat_ (s^−1^)	K_m_ (mM)	K_m_^H_2_O_2_^ (mM)	*k*_cat_/K_m_ (mM^−1^ s^−1^)	Reference
NR ^†^	28 ± 3	0.11 ± 0.01	31 ± 4	258	This work
TCP ^†^	70 ± 6	0.10 ± 0.02	94 ± 8	700	[43]

^†^ Phosphate buffer, pH 6.5.

## Data Availability

Raw data could be found at Data Repository https://doi.org/10.5281/zenodo.8106488, https://zenodo.org/record/8106488 (accessed on 22 June 2023).

## Supplementary Materials

The following supporting information can be downloaded at: https://www.mdpi.com/article/10.3390/ijms241311070/s1.
