# Supplementary material for: Dye Decolorization by a Miniaturized Peroxidase Fe-MimochromeVI*a
Source: Int J Mol Sci. 2023 Jul 4;24(13):11070. doi: 10.3390/ijms241311070 (PMC10342269; doi:10.3390/ijms241311070)
Supplement: Supplementary file 1 [file ijms-24-11070-s001.zip › ijms-2474471-supplementary.pdf]

International Journal of *Molecular Sciences*

MDPI

Supplementary Materials

Dye Decolorization by a Miniaturized Peroxidase Fe-MimochromeVI*a

Marco Chino [1,*], Salvatore La Gatta [1], Linda Leone [1], Maria De Fenza [1], Angela Lombardi [1], Vincenzo Pavone [1] and Ornella Maglio [1,2,*]

1 Department of Chemical Sciences, University of Napoli Federico II, Via Cintia, 80126 Napoli, Italy; salvatore.lagatta@unina.it (S.L.G.); linda.leone@unina.it (L.L.); maria.defenza@unina.it (M.D.F.); alombard@unina.it (A.L.); vincenzo.pavone@unina.it (V.P.)

2 Institute of Biostructures and Bioimaging (IBB), National Research Council (CNR), Via Pietro Castellino 111, 80131 Napoli, Italy

* Correspondence: marco.chino@unina.it (M.C.); ornella.maglio@unina.it (O.M.); Tel.: +39-081-674429 (M.C.); +39-081-674400 (O.M.)

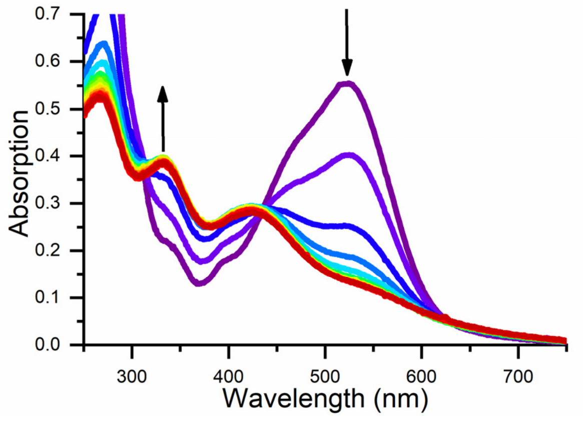

Figure S1. UV-visible spectroscopic changes upon mixing NR (0.06 mM), FeMC6*a (1 mM) and H_2O_2 (0.1 mM). The spectra were acquired at 25 °C in phosphate buffer (100 mM, pH 6.5), and collected every minute after addition of H_2O_2 (from violet to red), for a total time of 15 minutes.

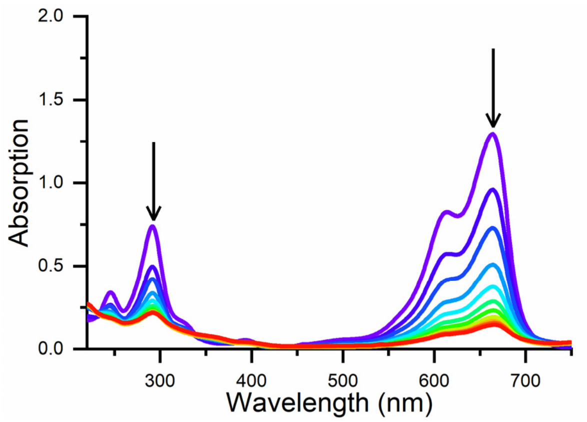

Figure S2. UV-visible spectroscopic changes upon mixing MB (12.9 mM), FeMC6*a (1 mM) and H_2O_2 (0.25 mM). The spectra were acquired at 25 °C in phosphate buffer (100 mM, pH 6.5), and collected every minute after addition of H_2O_2 (from violet to red), for a total time of 10 minutes.

Determination of molar extinction coefficient of the dyes

The molar absorptivity (ε) was determined for all four dyes using UV-vis spectroscopy. UV-vis spectra were recorded at room temperature in 100 mM phosphate buffer pH 6.5. Quartz cuvettes with a path length of 0.1 cm were used in all measurements. Wavelength scans were performed from 200 to 800 nm, with a 600 nm min^{-1} scan speed. All data were blank subtracted.

For all dyes UV-vis spectra were recorded at different concentrations and the absorbance values, at different wavelength (λ = 520 nm for NR, λ = 663 nm for MB, λ = 591 nm for BPB and λ = 580 nm for XO), were plotted as a function of dye concentration. The experimental data were fitted to a Lambert-Beer's law.

The following experimental conditions were used.

Neutral Red. Neutral Red stock solution was prepared dissolving 0.98 mg (3.39 mmol) of solid powder in 1.00 mL of phosphate buffer solution 100 mM (pH 6.5) (concentration 3.39 mM). Starting from this stock solution, four different diluted solutions were prepared (1.0 mL final volume) and then used to obtain the ε value at 520 nm. The experimental data were fitted to a Lambert-Beer's law, giving a ε_{520} = (9.47 ± 0.06) 10^3 M^{-1} cm^{-1}. The UV/vis absorption spectra and the plot of the absorbance at 520 nm as a function of NR concentration are reported in Figure S3.

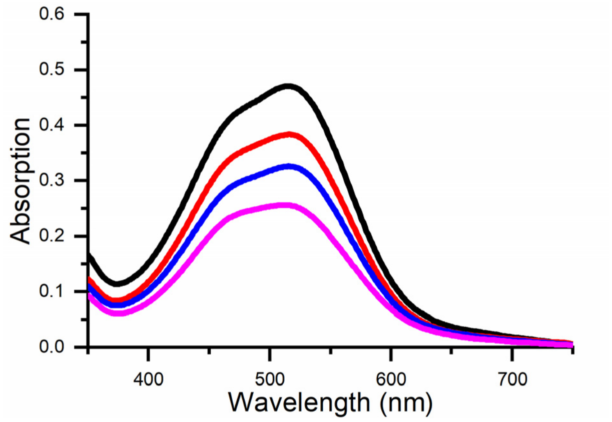

(a)

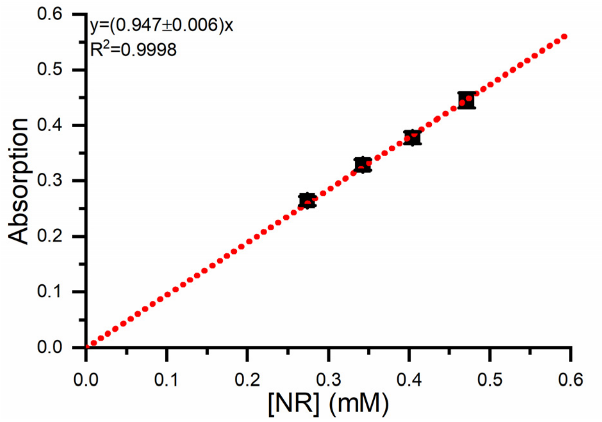

(b)

Figure S3. (**a**) Absorption spectra of NR solutions at different concentrations (0.471 mM, black; 0.404 mM, red; 0.343 mM blue; 0.274 mM magenta). (**b**) Plot of the absorbance at 520 nm as a function of NR concentration.

Methylene Blue. 0.83 mg (2.22 μmol) of MB were dissolved in 2.00 mL of phosphate buffer solution 100 mM (pH 6.5) (concentration 1.11 mM). This stock solution was used to prepare diluted samples, which were used to derive the ε value at 663 nm, ε_{663} = (10.05 ± 0.04) 10^4 M^{-1} cm^{-1}. The UV-vis absorption spectra and the plot of the absorbance at 663 nm as a function of MB concentration are reported in Figure S4.

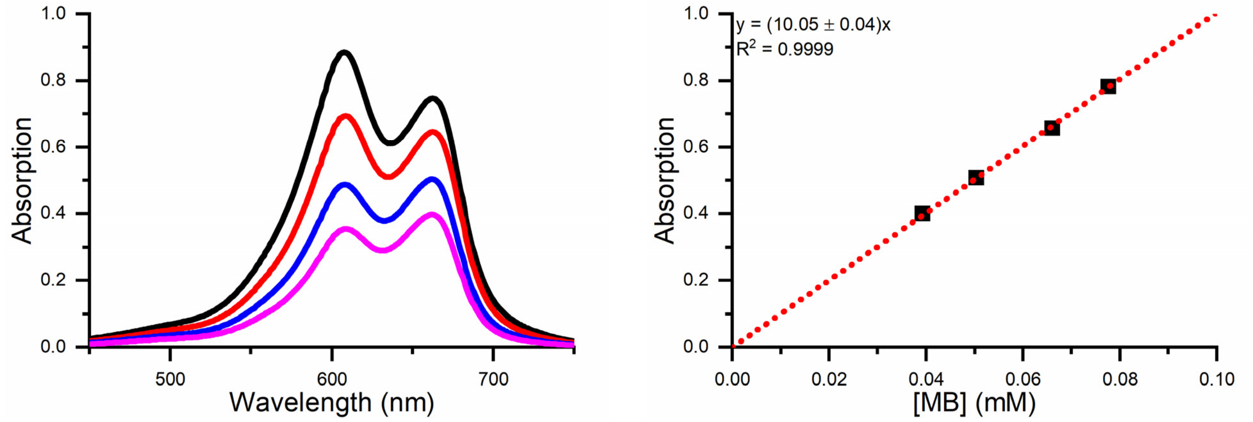

(a) **(b)**

Figure S4. (**a**) Absorption spectra of MB solutions at different concentrations (0.078 mM, black; 0.066 mM, red; 0.050 mM blue; 0.039 mM magenta). (**b**) Plot of the absorbance at 663 nm as a function of MB concentration.

Interestingly, the MB is characterized by two bands. The first absorption maximum is at 663 nm and relates to the monomer of methylene blue, while the second maximum (about 610 nm) can be attributed to a dimeric form.
It is well known that MB dye molecules, in water, self-aggregate to form, not only dimeric species but also aggregates of different orders, depending on the total MB concentration and the temperature, and these aggregates are characterized by absorption maximum ranging from 610 to 585 nm [1].

<u>*Xylenol Orange*</u>. A XO stock solution 1.33 mM was diluted with buffer to prepare different diluted samples. UV-vis spectra of these samples were then collected (Figure S5a) and the absorbance at 576 nm was plotted as a function of dye concentration to obtain the e value at 576 nm (Figure S5b). The fitting of experimental data gave the ε value ε_{576} = (0.896± 0.006) 10^4 M^{-1} cm^{-1}.

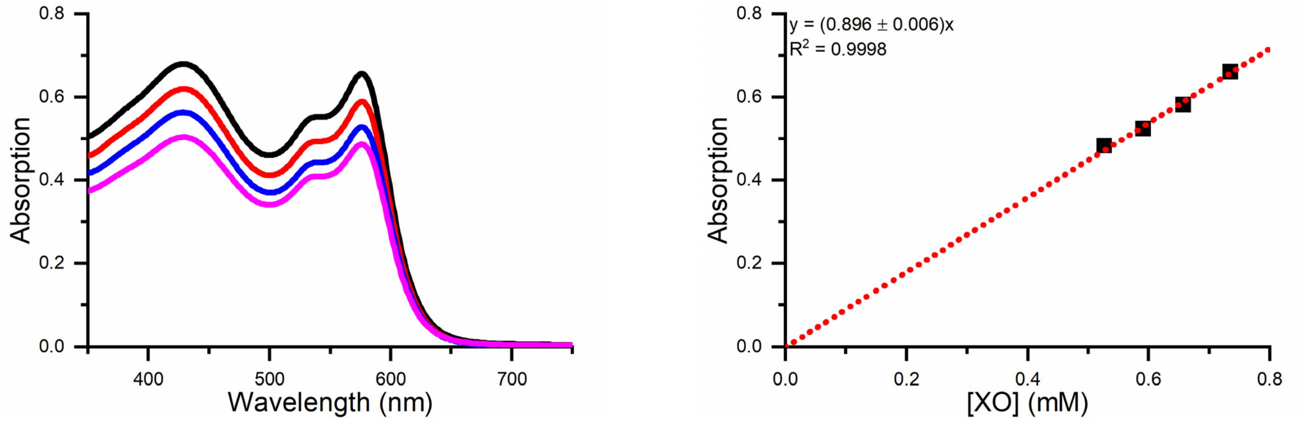

(a) **(b)**

Figure S5. (**a**) Absorption spectra of XO solutions at different concentrations (0.735 mM, black; 0.657 mM, red; 0.591 mM blue; 0.527 mM magenta). (**b**) Plot of the absorbance at 576 nm as a function of XO concentration.

<u>*Bromophenol Blue*</u>. Stock solution of BPB (0.68 mM) was prepared by dissolving 0.91 mg (1.36 mmol) of solid powder in 2.00 mL of phosphate buffer 100 mM (pH 6.5). Starting from this solution, four diluted solutions were further prepared and used to derive the ε value at 591 nm ε_{591} = (10.54±0.05) 10^4 M^{-1} cm^{-1}. The UV/vis absorption spectra and the plot of the absorbance at 591 nm as a function of BPB concentration are reported in Figure S6.

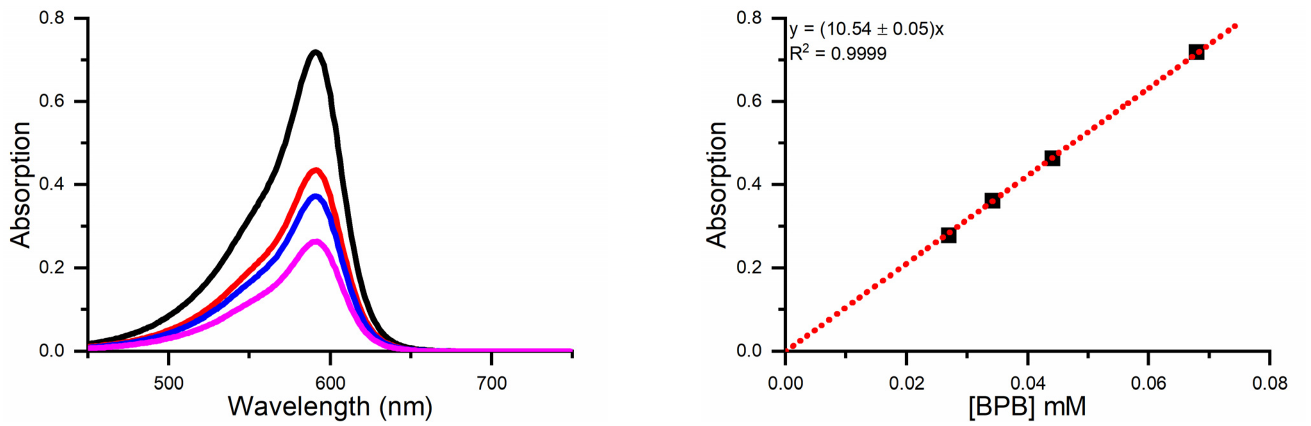

(a) (b)

Figure S6. (**a**) Absorption spectra of BPB solutions at different concentrations (0.068 mM, black; 0.044 mM, red; 0.034 mM blue; 0.027 mM magenta). (**b**) Plot of the absorbance at 591 nm as a function of BPB concentration.

Table S1 summarizes the main characteristics of the dyes used in this study.

Table S1. Concentration range and wavelength used for extinction coefficient determination of the dyes used in this study, ε values and percentage of dye color removal by FeMC6*a.

Substrate	Conc. Range (μM)	λ (nm)	ε ($M^{-1}cm^{-1}$)*	Decolorization level (%)
Neutral Red (NR)	274.1 – 470.9	520	9470	70
Methylene Blue (MB)	39.2 – 77.6	663	100500	95
Xylenol Orange (XO)	527.0 – 735.0	576	8960	82
Bromophenol Blue (BPB)	27.0 – 67.9	591	105400	18

*Phosphate buffer (0.1 M, pH 6.5)

References

1. Bergmann, K.; O'Konski, C.T. A Spectroscopic Study of Methylene Blue Monomer, Dimer, and Complexes with Montmorillonite. *J. Phys. Chem.* **1963**, *67*, 2169–2177, doi:10.1021/j100804a048.
